# Publisher Correction: Coupling of ocean redox and animal evolution during the Ediacaran-Cambrian transition

**DOI:** 10.1038/s41467-018-05540-7

**Published:** 2018-08-20

**Authors:** Dan Wang, Hong-Fei Ling, Ulrich Struck, Xiang-Kun Zhu, Maoyan Zhu, Tianchen He, Ben Yang, Antonia Gamper, Graham A. Shields

**Affiliations:** 1 MNR Key Laboratory of Isotope Geology, MNR Key Laboratory of Deep-Earth Dynamics, Institute of Geology, Chinese Academy of Geological Sciences, 100037 Beijing, China; 2 State Key Laboratory for Mineral Deposits Research, School of Earth Sciences and Engineering, Nanjing University, Nanjing, 210023 China; 3 Museum für Naturkunde, Leibniz Institute for Evolution and Biodiversity Science, 10115 Berlin, Germany; 4 Department of Earth Sciences, Freie Universität Berlin, Malteserstrasse 74-100, Haus D, 12249 Berlin, Germany; 5 State Key Laboratory of Palaeobiology and Stratigraphy, Center for Excellence in Life and Paleoenvironment, Nanjing Institute of Geology and Palaeontology, Chinese Academy of Sciences, 210008 Nanjing, China; 6 College of Earth Sciences, University of Chinese Academy of Sciences, 100049 Beijing, China; 7 School of Earth and Environment, University of Leeds, Leeds, LS2 9JT UK; 8 MNR Key Laboratory of Stratigraphy and Palaeontology, Institute of Geology, Chinese Academy of Geological Sciences, 100037 Beijing, China; 9 Department of Earth Sciences, University College London, Gower Street, London, WC1E 6BT UK

Correction to: *Nature Communications *10.1038/s41467-018-04980-5; published online: 03 July 2018

The original version of this Article incorrectly gave the second address in the list of affiliations as “State Key Laboratory of Palaeobiology and Stratigraphy & Center for Excellence in Life and Paleoenvironment, Nanjing Institute of Geology and Palaeontology, Chinese Academy of Sciences, 210008 Nanjing, China”, instead of the correct ‘State Key Laboratory for Mineral Deposits Research, School of Earth Sciences and Engineering, Nanjing University, Nanjing 210023, China”. This has been corrected in both the PDF and HTML versions of the Article.

