# Supplementary material for: Coupling of ocean redox and animal evolution during the Ediacaran-Cambrian transition
Source: Nat Commun. 2018 Jul 3;9:2575. doi: 10.1038/s41467-018-04980-5 (PMC6030108; doi:10.1038/s41467-018-04980-5)
Supplement: Supplementary file 1 — Supplementary Information [file 41467_2018_4980_MOESM1_ESM.pdf]

Coupling of ocean redox and animal evolution during the Ediacaran-Cambrian transition

Wang et al.

Supplementary Note

Supplementary Note 1. Lithology and data results of the Yanjia section in Zhejiang

The Yanjia section was deposited in a slope-deep basinal setting in the Lower Yangtze region (eastern part of the Yangtze block), which consists of Piyuancun Formation and the overlying Hetang Formation. The Piyuancun Formation is composed of organic-rich cherts and black shales, while the Hetang Formation comprises mainly black shales with phosphatic nodules and a Ni-Mo rich layer at the base (Supplementary Fig. 1). The results of bulk nitrogen ($\delta^{15}N$) and organic carbon ($\delta^{13}C_{org}$) isotopes, total organic carbon (TOC) and total nitrogen concentrations (TN) in the decarbonated samples from Yanjia sections are presented in Supplementary Data 2, together with TOC/N ratios (calculated from the measured TOC and TN contents on an atomic basis). $\delta^{15}N$ and $\delta^{13}C_{org}$ data are plotted against depth in Supplementary Fig. 1.

The $\delta^{15}N_{bulk}$ values of the lower-middle part of the Piyuancun Formation range from +3.3 to +6.3‰, and then decline to a minimum of +1.0‰ and a lower average of +2.6‰ in the upper part of the Piyuancun Formation. The $\delta^{13}C_{org}$ values are relatively high at the base of Piyuancun Formation, with a highest value of –25.9‰, and then show a sharp negative excursion in the immediate overlying layer to –32.8‰. After a short increase to –30.1‰, the $\delta^{13}C_{org}$ values show a mild but constant decreasing trend from –30.1‰ to –32.2‰ in the mid–upper Piyuancun Formation. In the Hetang Formation, the $\delta^{15}N_{bulk}$ dramatically decreases to below 0‰, with the nadir value (–6.3‰) of the whole section occurring at the basal part of this formation, alongside a negative $\delta^{13}C_{org}$ excursion with the nadir value of –34.3‰. Upwards, the $\delta^{15}N_{bulk}$ values stay relatively constant around +5.5‰ before declining to –0.7‰, while the $\delta^{13}C_{org}$ recovers to –31.0‰ keeps relatively constant around this value at the upper part of the Hetang Formation.

The primary N isotopic composition preserved in the ancient sediments may have been modified in early and/or late burial diagenesis and metamorphism. The plot of TN–TOC shows a significant linear correlation (Supplementary Fig. 2a), suggesting that the nitrogen originated from primary organic matter[1]. Although the TOC/N ratios are high with an average of 71.6 which probably resulted from the N loss during the diagenesis, the fact that plots of $\delta^{15}N$–TOC, $\delta^{15}N$–TN and $\delta^{15}N$–TOC/N exhibit no linear correlations (Supplementary Fig. 2b, 2c, 2d), suggesting that the primary $\delta^{15}N_{bulk}$ values have not been

significantly modified. Published iron species data in the studied area demonstrated a predominance of anoxic with intermitted euxinic environment during the Ediacaran–Cambrian transition. Anaerobic degradation of organic matter is suggested to retain the initial $\delta^{15}N$ value or decrease $\delta^{15}N$ only to a slight extent, on the order of 2–3‰[2, 3, 4, 5, 6]. Thus the large variation of $\delta^{15}N_{bulk}$ values (~12‰) at Yuanjia section is apparently not the case of organic matter decomposition under anoxic condition. The kerogen H/C ratios from the Piyuancun and Hetang formations at neighboring Diben section range from 0.19 to 0.65, averaging 0.3[7], suggesting that the studied area has not experienced significant metamorphism above greenschist–amphibolite facies[8, 9]. This conclusion is consistent with petrographic observation of the studied Yanjia section which shows no apparently metamorphic feature. Metamorphism could increase $\delta^{15}N$ values in phyllosilicates while $\delta^{15}N$ values in organic matter become lighter[10]. However, the isotopic effect of nitrogen loss from the system has been suggested to be ~1-2‰ units at greenschist facies and ~3–4‰ units at amphibolite facies[10, 11]. Thus nitrogen isotopic shifts if any caused by diagenesis and low-grade metamorphism should be expressed only in a limited extant and consistently throughout the section in such a short stratigraphic interval. Therefore, the $\delta^{15}N$ variations observed at Yanjia section record the primary signals of the late Ediacaran–early Cambrian basin in the lower Yangtze region.

Supplementary Note 2. Stratigraphic correlation on the Yangtze block

The stratigraphic correlation of deep-water strata with shallow-water equivalents could be constrained by reliable fossil records, accurate radiometric dating and carbon isotopic data ($\delta^{13}C_{carb}$ and $\delta^{13}C_{org}$) during the Ediacaran–Cambrian transition. During the latest Ediacaran, carbonate deposits of Dengying Formation widely developed in shallow-water platform and changed basinward into cherts and shale of the Liuchapo/Piyuancun/Laobao Formation in deep-water settings (Supplementary Fig. 3). A black shale horizon underlying the Dengying (and its correlative units) is widespread in almost all paleoenvironment in the Yangtze region and can be used as a reliable stratigraphic marker[12]. A U-Pb zircon age of 551.1 ± 0.7 Ma from the very top of this marker horizon has been obtained[13] (Supplementary Fig. 3), and can be considered as the beginning of Dengying deposition and its correlative units (Liuchapo, Piyuancun or

Laobao formations). In shallow platform to slope settings, the Precambrian–Cambrian (Pc/C) boundary was placed at the base of the siliceous dolostone and/or chert deposits (Zhujianqing/Laobao/Liuchapo Formation) overlying the Dengying Formation, on the basis of the biostratigraphy of small shelly fossils (SSFs)[14, 15, 16] (Supplementary Fig. 3) and occurrence of prounced negative carbon isotopic anomalies (BAsal Cambrian Carbon isotope Excursion, named BACE, ref. 17, Supplementary Fig. 4) . This conclusion is consistent with the zircon U-Pb age of 542.1 ± 5.0 Ma beneath chert deposits in northeastern Guizhou[18] (Supplementary Fig. 3). However, in the slope to basinal settings where the carbonate deposits of Dengying Formation was thoroughly replaced by cherts, the Pc/C boundary could be constrained by negative $\delta^{13}C_{org}$ excursions at mid-upper Liuchapo/Piyuancun Formation (Supplementary Fig. 4), which is further supported by a zircon U-Pb age of 542.6 ± 3.7 Ma at mid-upper part of cherts deposition in northeastern Guizhou[18] (Supplementary Fig. 3).

In the shallow platform, the lower-middle Zhujiaqing Formation in eastern Yunan belongs to the Cambrian Fortunian stage, while the upper part was deposited in the early Cambrian Stage 2 (Supplementary Fig. 3), characterized by a large carbon positive excursion (ZHUjiaqing Carbon isotope Excursion, named as ZHUCE, ref. 17, Supplementary Fig. 4). In deeper shelf region, basal units of the black-shale deposits (Niutitang Formation) in western Hunan contain rare SSFs *Protohertzina anabarica–Kaiyangites novilis* Assemblage Zone of Cambrian Fortunian Stage[16, 19, 20] (Supplementary Fig. 3). And K-bentonite layers in the upper Liuchapo Formation of western Hunan and northeastern Guizhou could be equivalent to that in the middle Zhujiaqing Formation in Eastern Yunnan, with similar zircon U-Pb ages 536.3 ± 5.5 Ma, 536 ± 5 Ma and 539.4 ± 2.9 Ma, respectively[21, 22] (Supplementary Fig. 3). However, carbon positive excursions (ZHUCE) are not clearly identified at the deep-water sections. Thus the Cambrian Fortunian Stage–early Stage 2 successions in the marginal shelf to basinal areas either is condensed or has sedimentary hiatus in the Yangtze region[23].

Overlying the Liuchapo Formation and its correlative units, the homogeneous deposition of black shales referred to Niutitang (or Xiaoyanxi, Zhalagou, Hetang) Formation widely developed in marginal shelf to basinal settings (Supplementary Fig. 3), representing a major transgression event. Widespread K-bentonite beds at the basal part

of black-shale deposits (Niutitang and its equivalent formations) in deep-water realms could be correlated with that in the basal Shiyantou Formation in northeastern Yunnan on the basis of their mineralogical and geochemical characteristics[24], although their zircon U–Pb dating data are not fully consistent: 526.5 ± 1.1 Ma in Yunnan[22], 518 ± 5 Ma[25], 522.7 ± 4.9 Ma[26], 522.3 ± 3.7 Ma[18], 524.2 ± 5.1 Ma[18], and 532.3 ± 0.7 Ma[27] in Guizhou (Supplementary Fig. 3). Overlying this K-bentonite beds, a widely distributed Ni–Mo sulfide layer in the lower part of the black-shale deposits can be used as a correlation marker in the Yangtze region[12, 28, 29], which could correlate with the Ni-Mo enrichments in the basal Yu'anshan Formation at Xiaotan section in Yunnan[28] (Supplementary Fig. 3). Xu, Lehmann et al. (2011) have reported a Re-Os isochron age of 521 ± 5 Ma for this Ni-Mo sulfide layer[30] (Supplementary Fig. 3), suggesting that the Ni-Mo layer deposition belongs to the late Cambrian Stage 2. In addition, the negative $\delta^{13}C_{carb}$ and $\delta^{13}C_{org}$ excursions widely occurred from platform to basinal realms within or near the Ni-Mo layer (SHIyantou Carbon isotope Excursion, named as SHICE, ref. 17, Supplementary Fig. 4).

Above the Ni-Mo layer, prevalent positive $\delta^{13}C_{carb}$ or $\delta^{13}C_{org}$ excursion (Cambrian Arthropod Radiation isotope Excursion, named as CARE, ref. 17) in the Cambrian Stage 3 could also be a mark of the stratigraphic correlation from platform to basinal realms[31, 32] (Supplementary Fig. 4). Trilobite *Hunanocephalus* and *Tusnyidicus* has been reported in the upper part of Niutitang Formation in Hunan and Guizhou, respectively[33] (Supplementary Fig. 3), which suggests that most of the black-shale deposits (Niutitang and its equivalent formations) deposited during the Cambrian Stage 3. Unfortunately, the rare distribution of the trilobites in deep-water sections makes the correlation between Yu'anshan Formation and the upper Niutitang/Xiaoyanxi /Zhalagou/Hetang formations ambiguous.

Supplementary Note 3. Stratigraphic correlation between the Kazakhstan and Yangtze blocks

The Paleo-Kazakhstan has been suggested to be an island arc, which was separated by a narrow oceanic basin from the Yangtze block during the late Neoproterozoic and early Cambrian, supported by their similarities in ichnofacies and depositional regimes[34].

The Ediacaran–early Cambrian successions developped in the Malyi Karatau area of the southern Kazakhstan with well-preserved fossil assemblages, which consist of the Kyrshabakty, Chulaktau and Shabakty formations in ascengding order and represent shallow platform environments[35] (Supplementary Fig. 5). The upper part of the Kyrshabakty Formation is composed of dolostone and referred to the Berkuty Member, and the Chulaktau Formation is divided into Aksai, Karatau and Ushbas members, which comprise mainly cherty dolostone, cherty dolostone with phosphorite layer and Fe-Mn rich dolostone, respectively (Supplementary Fig. 5). The overlying Shabakty Formation is characterized by dolo- and limestones[34] (Supplementary Fig. 5). Previous studies has proposed that the uppermost part of Berkuty Member already belongs to the Cambrian Fortunian Stage based on the its host of *Anabaritestrisulcatus*-*Protohertzinaanabarica* Assemblage Zone[34, 35] (Supplementary Fig. 5). Upwards, the Chulaktau Formation contain the *Pseudorthotheca costata* Zone at the base of the Karatau Member and the *Bercutia cristata* Zone near the top of the Ushbas Member, which are roughly time equivalent with *Watsonella crosbyi* to *Sinosachites flabelliformis–Tannuolina Zhangwetangi* Assemblage Zone on the Yangtze platform[34] (Supplementary Fig. 5). Thus the lower Chulaktau Formation (Aksai Member) is probably time equivalent with the lower-middle Zhujiaqing Formation of South China, belonging to the Cambrian Fortunian Stage, while the middile-upper part of Chulaktau Formation (Karatau and Ushbas members) could correlate with the upper Zhujiaqing and Shiyantou formations, belonging to the Cambrian Stage 2 (Supplementary Fig. 5, refs 34, 35). Unfortunately, there is a disconformity between Chulaktau Formation and the overlying Shabakty Formation, as well as a biostratigraphic gap with unknow duation[34]. The Shabakty Formation contain *Rhombicorniculum cancellatum* Assemblage Zone and the first trilobite zones (*Ushbaspis limbata*- and *Hebediscus orientalis* Zones), implying that Shabakty Formation belongs to Cambrian Stage 3 (Supplementary Fig. 5, ref. 34). The *R. cancellatum* Taxon Range Zone has also been identified on the Yangtze block[16], and its host strata possibly correlate to the upper Yu'anshan Formation at the shallow platform in Yunan[16, 34] (Supplementary Fig. 5).

Supplementary Note 4. Student's t-test for statistical differences between positive and negative $\delta^{15}N$ excursions

In order to statistically determine whether the inferred nitrogen isotopic excursions are indeed significant, a student's t-test was performed in five groups (intervals I–II, intervals II–III, intervals III–IV, intervals IV–V and intervals V–VI). Our null hypothesis is that there are no differences between positive and negative $\delta^{15}N$ excursions. When p-value is lower than 0.05, the null hypothesis can be rejected. The results show that p-values are much lower than 0.05 ($<10^{-4}$, Supplementary Fig. 6). In other words, we can be reasonably confident that differences between positive and negative $\delta^{15}N$ values are highly significance.

Supplementary figures and legends

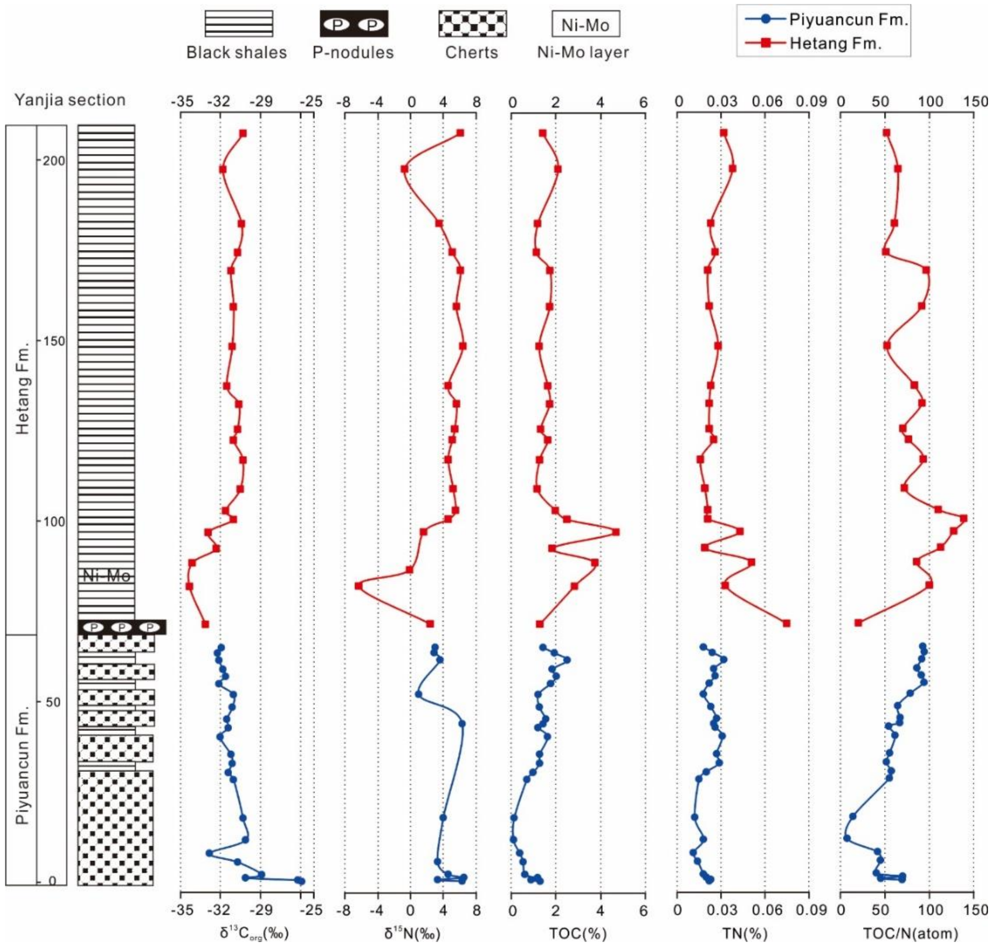

Supplementary Fig. 1 Profiles of nitrogen and organic carbon isotopes, TOC and TN contents and TOC/N ratios for the Yanjia section.

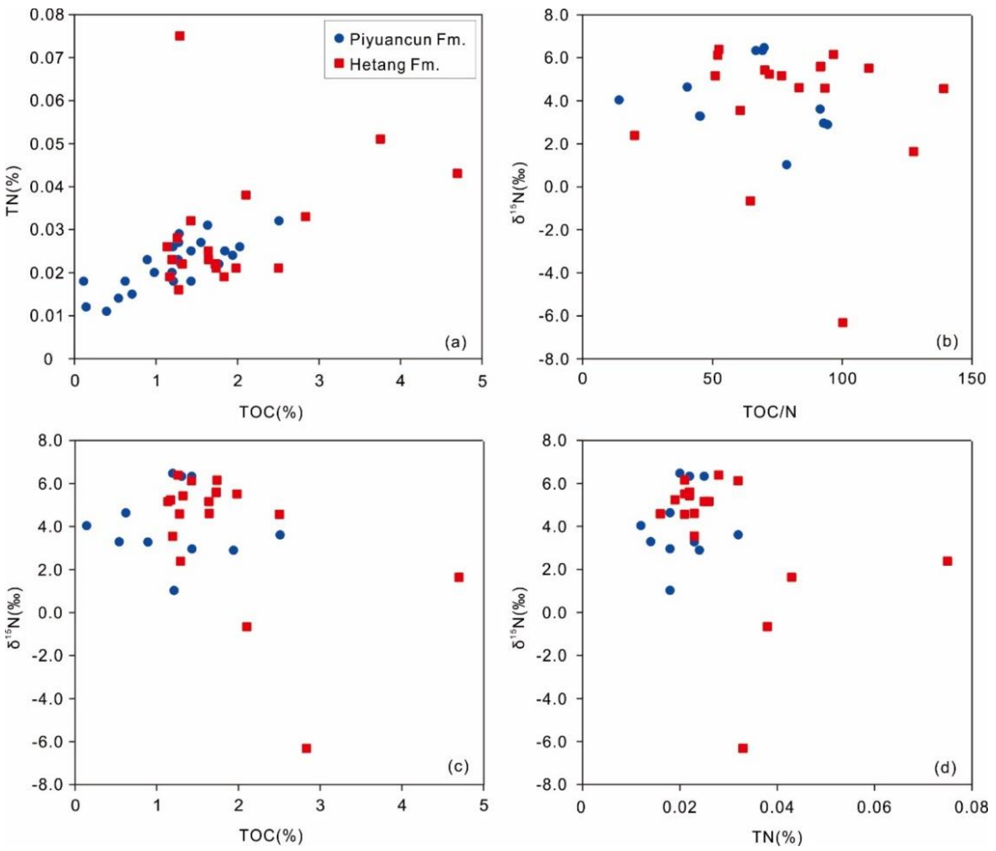

Supplementary Fig. 2 Cross plots of (a) TN versus TOC, (b) $\delta^{15}N$ versus TOC/N ratios, (c) $\delta^{15}N$ versus TOC and (d) $\delta^{15}N$ versus TN for the Yanjia section

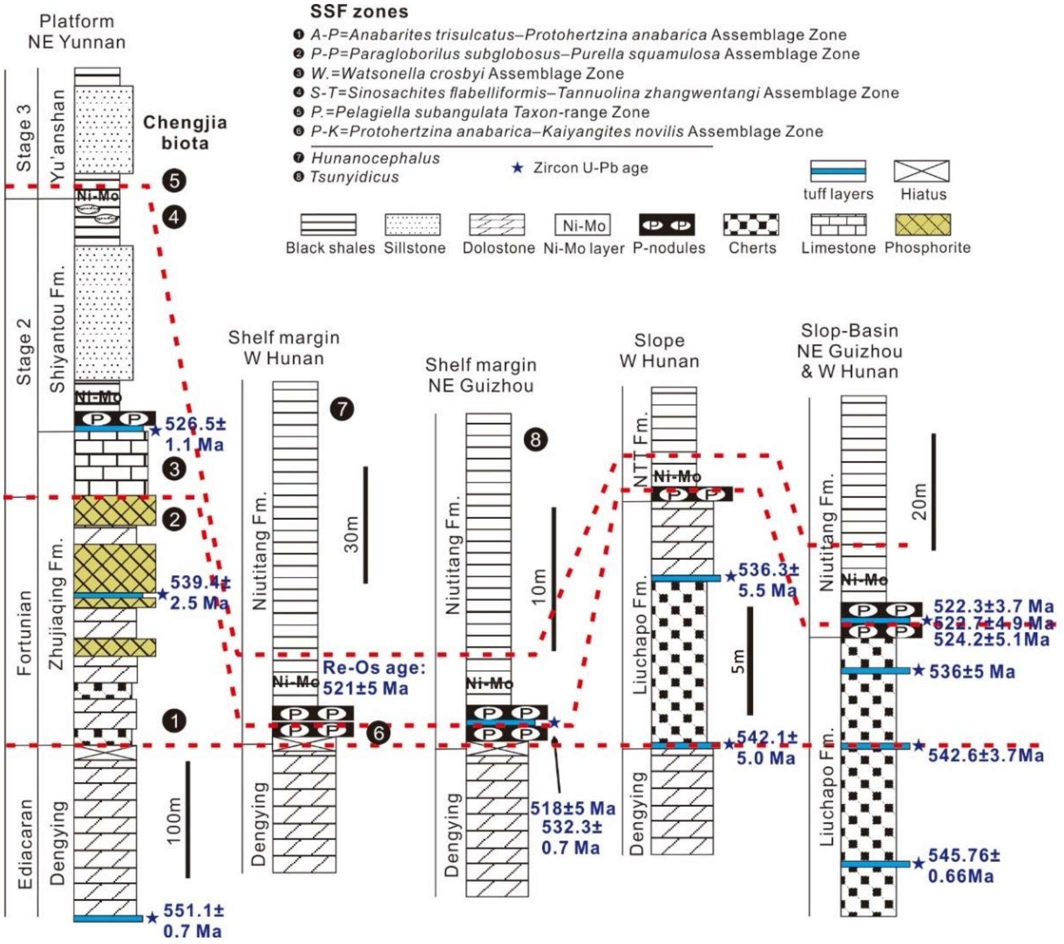

Supplementary Fig. 3 Stratigraphic correlation, fossil records and radiometric ages on Yangtze block. Fossil records are mainly from refs 16, 19, 20, 33, 36, 37, 38. Zircon U–Pb ages are from refs 13, 18, 21, 22, 26, 27, 39. Re-Os age is from ref. 30.

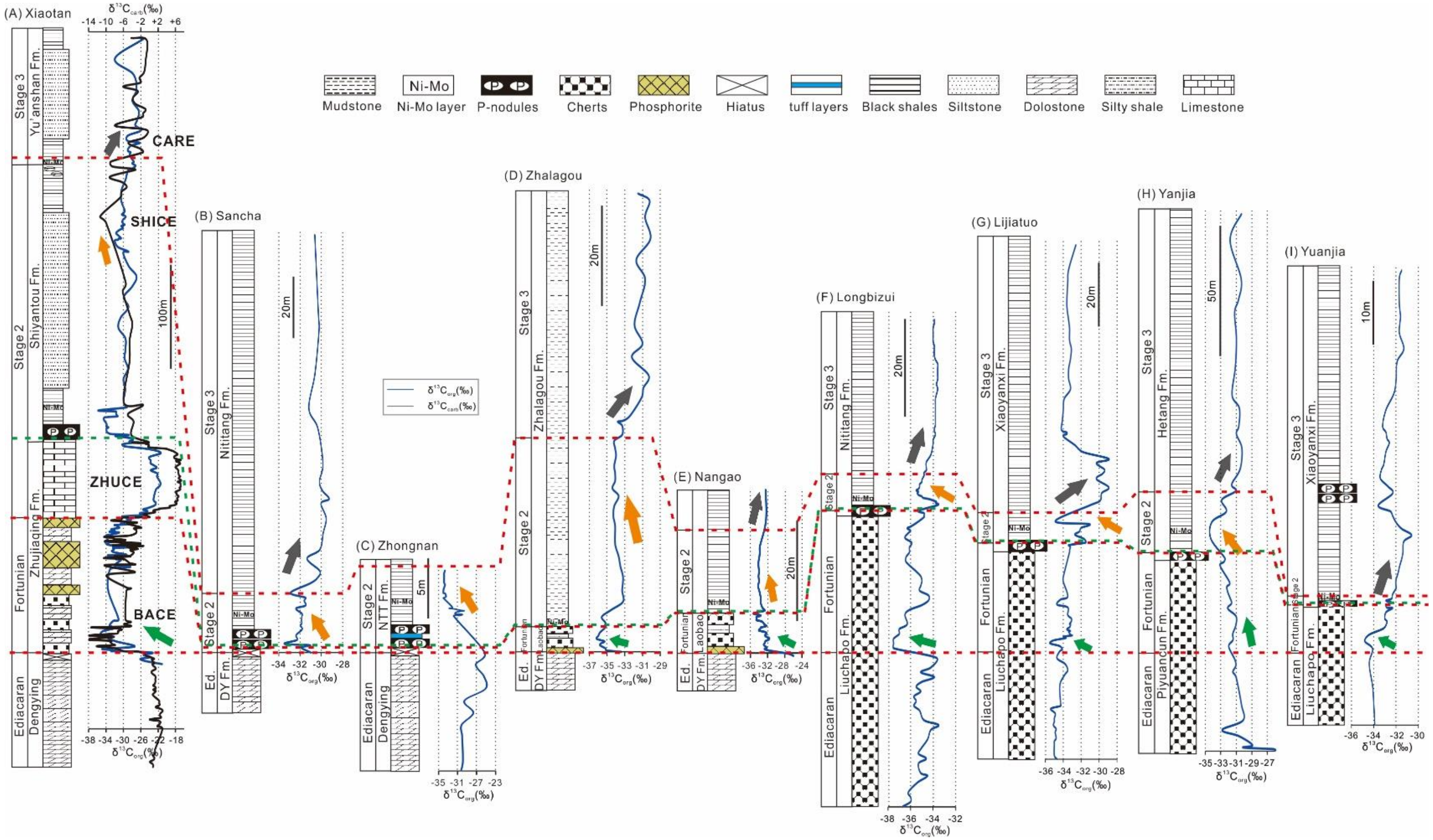

Supplementary Fig. 4 Carbon isotope stratigraphic correlation of (A) Xiaotan section in Yunnan[15, 40], (B) Sancha section in Hunan[41], (C) Zhongnan section in Guizhou[42], (D) Zhalagou sectionmin Guizhou[32], (E) Nangao section in Guizhou[43], (F) Longbizui section in Hunan[31], (G) Lijiatuo section in Hunan[44], (H) Yanjia section in Zhejiang (this study), (I) Yuanjia section in Hunan[31]

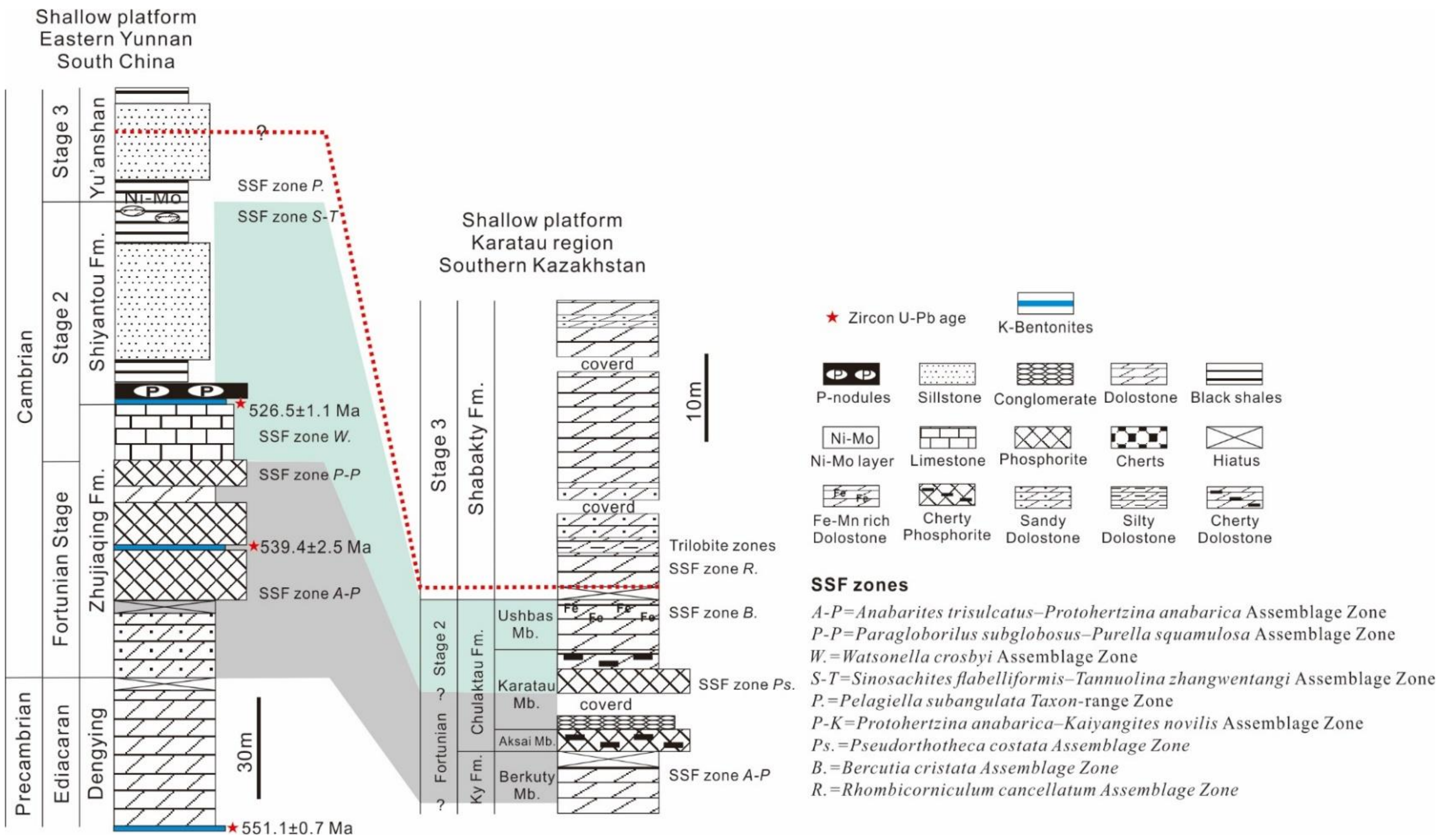

Supplementary Fig. 5 Stratigraphic correlation between the Kazakhstan and Yangtze blocks. Fossil records are mainly from refs 34, 36, 37. Zircon U–Pb ages are from refs 13, 22

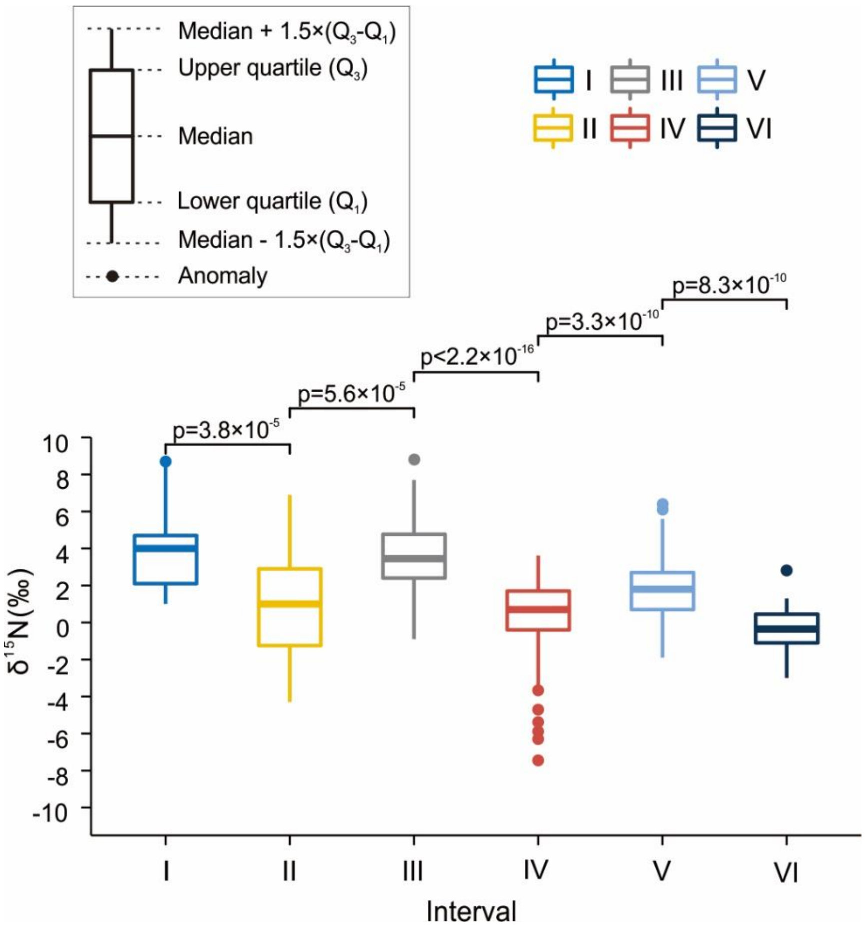

Supplementary Fig. 6 Box and whisker plots of $\delta^{15}N$ values in different time intervals, with p-values of student's t-test performed in five groups (intervals I–II, intervals II–III, intervals III–IV, intervals IV–V and intervals V–VI).

Supplementary References

1. Calvert, S. Beware intercepts: interpreting compositional ratios in multi-component sediments and sedimentary rocks. *Org Geochem* **35**, 981-987 (2004).
2. Altabet, M. A*., et al.* The nitrogen isotope biogeochemistry of sinking particles from the margin of the Eastern North Pacific. *Deep-sea research Part I, Oceanographic research papers* **46**, 655-679 (1999).
3. Junium, C. K. & Arthur, M. A. Nitrogen cycling during the Cretaceous, Cenomanian-Turonian oceanic anoxic event II. *Geochemistry, Geophysics, Geosystems* **8**, Q03002 (2007).
4. Möbius, J., Lahajnar, N. & Emeis, K.-C. Diagenetic control of nitrogen isotope ratios in Holocene sapropels and recent sediments from the Eastern Mediterranean Sea. *Biogeosciences Discussions* **7**, 1131-1165 (2010).
5. Higgins, M. B., Robinson, R. S., Carter, S. J. & Pearson, A. Evidence from chlorin nitrogen isotopes for alternating nutrient regimes in the Eastern Mediterranean Sea. *Earth Planet Sc Lett* **290**, 102-107 (2010).
6. Lehmann, M. F., Bernasconi, S. M., Barbieri, A. & McKenzie, J. A. Preservation of organic matter and alteration of its carbon and nitrogen isotope composition during simulated and in situ early sedimentary diagenesis. *Geochim Cosmochim Acta* **66**, 3573-3584 (2002).
7. Yuan, Y*., et al.* Redox condition during Ediacaran–Cambrian transition in the Lower Yangtze deep water basin, South China: constraints from iron speciation and δ 13Corg in the Diben section, Zhejiang. *Chinese Science Bulletin* **59**, 3638-3649 (2014).
8. Strauss, H., Des Marais, D. J., Hayes, J. M. & Summons, R. E. Concentrations of organic carbon and maturities and elemental compositions of kerogens. *The Proterozoic Biosphere, A Multidisciplinary Study*, 95-99 (1992).
9. Hayes, J. M., Wedeking, K. W. & Kaplan, I. R. Precambrian organic geochemistry-Preservation of the record. In: *Earth's earliest biosphere: Its origin and evolution* (ed. Schopf, J.W.) 93-134 (Princeton University Press, 1983).
10. Stüeken, E. E., Zaloumis, J., Meixnerová, J. & Buick, R. Differential metamorphic

effects on nitrogen isotopes in kerogen extracts and bulk rocks. *Geochim Cosmochim Acta* **217**, 80-94 (2017).

11. Thomazo, C. & Papineau, D. Biogeochemical Cycling of Nitrogen on the Early Earth. *Elements* **9**, 345-351 (2013).
12. Zhu, M.*, et al.* Sinian-Cambrian stratigraphic framework for shallow-to deep-water environments of the Yangtze Platform: an integrated approach. *Prog Nat Sci* **13**, 951-960 (2003).
13. Condon, D.*, et al.* U-Pb ages from the neoproterozoic Doushantuo Formation, China. *Science* **308**, 95-98 (2005).
14. Shen, Y. & Schidlowski, M. New C isotope stratigraphy from southwest China: Implications for the placement of the Precambrian-Cambrian boundary on the Yangtze Platform and global correlations. *Geology* **28**, 623-626 (2000).
15. Zhou, C., Zhang, J., Li, G. & Yu, Z. Carbon and oxygen isotopic record of the Early Cambrian from the Xiaotan Section, Yunnan, south China. *Scientia Geologica Sinica* **32**, 201-211 (1997).
16. Steiner, M.*, et al.* Neoproterozoic to early Cambrian small shelly fossil assemblages and a revised biostratigraphic correlation of the Yangtze Platform (China). *Palaeogeography, Palaeoclimatology, Palaeoecology* **254**, 67-99 (2007).
17. Zhu, M. Y., Strauss, H. & Shields, G. A. From snowball earth to the Cambrian bioradiation: Calibration of Ediacaran-Cambrian earth history in South China. *Palaeogeogr Palaeocl* **254**, 1-6 (2007).
18. Chen, D.*, et al.* New U–Pb zircon ages of the Ediacaran–Cambrian boundary strata in South China. *Terr Nova* **27**, 62-68 (2015).
19. Steiner, M.*, et al.* Lower Cambrian small shelly faunas from Zhejiang (China) and their biostratigraphical implications. *Prog Nat Sci* **13**, 852-860 (2003).
20. Yang, B.*, et al.* Chert-hosted small shelly fossils: expanded tool of biostratigraphy in the Early Cambrian. *GFF* **136**, 303-308 (2014).
21. Chen, D. Z.*, et al.* Hydrothermal venting activities in the Early Cambrian, South China: Petrological, geochronological and stable isotopic constraints. *Chem Geol* **258**, 168-181 (2009).

22. Compston, W., *et al.* Further SHRIMP geochronology on the early Cambrian of South China. *Am J Sci* **308**, 399-420 (2008).
23. Corsetti, F. A. & Hagadorn, J. W. Precambrian-Cambrian transition: Death Valley, United States. *Geology* **28**, 299-302 (2000).
24. Zhou, M., Luo, T., Huff, W. D. & Liu, S. Prominent Lower Cambrian K-Bentonites In South China: Distribution, Mineralogy, and Geochemistry. *J Sediment Res* **84**, 842-853 (2014).
25. Wang, X., Shi, X., Jiang, G. & Zhang, W. New U–Pb age from the basal Niutitang Formation in South China: Implications for diachronous development and condensation of stratigraphic units across the Yangtze platform at the Ediacaran–Cambrian transition. *J Asian Earth Sci* **48**, 1-8 (2012).
26. Zhou, M. Z., *et al.* SHRIMP U-Pb zircon age of tuff at the bottom of the Lower Cambrian Niutitang Formation, Zunyi, South China. *Chinese Science Bulletin* **53**, 576-583 (2008).
27. Jiang, S.-Y., *et al.* Early Cambrian ocean anoxia in south China. *Nature* **459**, E5-E6 (2009).
28. Och, L. M., *et al.* Redox changes in Early Cambrian black shales at Xiaotan section, Yunnan Province, South China. *Precambrian Res* **225**, 166-189 (2013).
29. Jiang, G., *et al.* The origin of decoupled carbonate and organic carbon isotope signatures in the early Cambrian (ca. 542–520Ma) Yangtze platform. *Earth Planet Sc Lett* **317**, 96-110 (2012).
30. Xu, L. G., *et al.* Re-Os Age of Polymetallic Ni-Mo-PGE-Au Mineralization in Early Cambrian Black Shales of South China-A Reassessment. *Econ Geol* **106**, 511-522 (2011).
31. Guo, Q., *et al.* High resolution organic carbon isotope stratigraphy from a slope to basinal setting on the Yangtze Platform, South China: Implications for the Ediacaran-Cambrian transition. *Precambrian Res* **225**, 209-217 (2013).
32. Cai, C., *et al.* Marine C, S and N biogeochemical processes in the redox-stratified early Cambrian Yangtze ocean. *J Geol Soc* **172**, 390-406 (2015).
33. Steiner, M., Zhu, M., Zhao, Y. & Erdtmann, B.-D. Lower Cambrian burgess shale-

type fossil associations of south China. *Palaeogeography, Palaeoclimatology, Palaeoecology* **220**, 129-152 (2005).

34. Weber, B., Steiner, M., Evseev, S. & Yergaliev, G. First report of a Meishucun-type early Cambrian (Stage 2) ichnofauna from the Malyi Karatau area (SE Kazakhstan): Palaeoichnological, palaeoecological and palaeogeographical implications. *Palaeogeography, Palaeoclimatology, Palaeoecology* **392**, 209-231 (2013).
35. Missarzhevsky, V. & Mambetov, A. Stratigraphy and fauna of Cambrian and Precambrian boundary beds of Maly Karatau. *Trudy Akademii Nauka SSSR, Moscow* **326**, (1981).
36. Li, G. & Xiao, S. Tannuolina and Micrina (Tannuolinidae) from the Lower Cambrian of eastern Yunnan, South China, and their scleritome reconstruction. *J Paleontol* **78**, 900-913 (2004).
37. Yang, B., Steiner, M., Li, G. & Keupp, H. Terreneuvian small shelly faunas of East Yunnan (South China) and their biostratigraphic implications. *Palaeogeography, Palaeoclimatology, Palaeoecology* **398**, 28-58 (2014).
38. Yang, B*., et al.* Transitional Ediacaran–Cambrian small skeletal fossil assemblages from South China and Kazakhstan: Implications for chronostratigraphy and metazoan evolution. *Precambrian Res* **285**, 202-215 (2016).
39. Yang, C., Zhu, M., Condon, D. J. & Li, X.-H. Geochronological constraints on stratigraphic correlation and oceanic oxygenation in Ediacaran-Cambrian transition in South China. *J Asian Earth Sci* **140**, 75-81 (2017).
40. Cremonese, L*., et al.* Marine biogeochemical cycling during the early Cambrian constrained by a nitrogen and organic carbon isotope study of the Xiaotan section, South China. *Precambrian Res* **225**, 148-165 (2013).
41. Wang, D*., et al.* Marine redox variations and nitrogen cycle of the early Cambrian southern margin of the Yangtze Platform, South China: Evidence from nitrogen and organic carbon isotopes. *Precambrian Res* **267**, 209-226 (2015).
42. Cremonese, L*., et al.* Nitrogen and organic carbon isotope stratigraphy of the Yangtze Platform during the Ediacaran–Cambrian transition in South China.

Palaeogeography, Palaeoclimatology, Palaeoecology **398**, 165-186 (2014).

43. Yang, X. L., Zhu, M. Y., Guo, Q. J. & Zhao, Y. L. Organic carbon isotopic evolution during the Ediacaran-Cambrian transition interval in eastern Guizhou, South China: Paleoenvironmental and stratigraphic implications. *Acta Geol Sin-Engl* **81**, 194-203 (2007).
44. Guo, Q. J., *et al.* Carbon isotopic evolution of the terminal neoproterozoic and early Cambrian: Evidence from the Yangtze platform, South China. *Palaeogeogr Palaeocl* **254**, 140-157 (2007).
45. Zhang, J., *et al.* Heterogenous oceanic redox conditions through the Ediacaran-Cambrian boundary limited the metazoan zonation. *Scientific Reports* **7**, 8550 (2017).
46. Gamper, A. *Global trends in nutrient dynamics during the Ediacaran-Cambrian period as revealed by nitrogen and carbon isotope trends.* Doctoral thesis, Freie Universität Berlin, Germany (2014).
