# Supplementary material for: Coupling of ocean redox and animal evolution during the Ediacaran-Cambrian transition
Source: Nat Commun. 2018 Jul 3;9:2575. doi: 10.1038/s41467-018-04980-5 (PMC6030108; doi:10.1038/s41467-018-04980-5)
Supplement: Supplementary file 3 — Description of Additional Supplementary Files [file 41467_2018_4980_MOESM3_ESM.pdf]

Description of Additional Supplementary Files

File Name: Supplementary Data 1
Description: Time-averaged N-isotope data for late Ediacaran–early Cambrian successions.

File Name: Supplementary Data 2
Description: Organic C- and N- isotope and correlative data for the Yanjia section in Zhejiang, South China.
